# Clinical Considerations for the Integration of Adjuvant Olaparib into Practice for Early Breast Cancer: A Canadian Perspective

**DOI:** 10.3390/curroncol30080556

**Published:** 2023-08-17

**Authors:** Jan-Willem Henning, Jean-François Boileau, Larissa Peck, Tom McFarlane

**Affiliations:** 1 Tom Baker Cancer Centre, University of Calgary, Calgary, AB T2N 4N2, Canada; 2 Segal Cancer Centre, Jewish General Hospital, McGill University, Montreal, QC H3T 1E2, Canada; jean-francois.boileau@mcgill.ca; 3 Princess Margaret Cancer Centre, Toronto, ON M5G 2M9, Canada; larissa.peck@uhn.ca; 4 Sunnybrook Health Sciences Centre, Toronto, ON M4N 3M5, Canada; tmcfarla@uwaterloo.ca

**Keywords:** adjuvant therapy, BRCA, capecitabine, early-stage breast cancer, genetic testing, olaparib, poly(ADP-ribose) polymerase inhibitors, pembrolizumab

## Abstract

With the recent Health Canada approval of olaparib for high-risk, HER2-negative early breast cancer, physicians are now facing the practical challenges of integrating olaparib into current management of triple-negative breast cancer (TNBC) and HR-positive, HER2-negative (HR+/HER2−) early breast cancer. This review provides perspectives on some of the challenges related to identification of olaparib candidates, with a focus on the latest guidance for germline BRCA testing and considerations regarding high-risk disease definitions. Updated treatment pathways are explored for both disease states, including other adjuvant treatment options such as pembrolizumab, capecitabine, and abemaciclib. Gaps in the current literature regarding the sequential or combined use of these adjuvant therapies are noted and future, potentially informative, studies are briefly examined.

## 1. Introduction

Pathogenic or likely pathogenic variants in *BRCA1* and *BRCA2*, commonly referred to as mutations in BRCA, are associated with an increased risk of breast, ovarian, prostate, and pancreatic cancer. Germline BRCA (gBRCA) mutations can explain up to 10% of cases of breast cancer and occur most frequently in patients with a breast cancer diagnosis at or before the age of 40, a triple-negative breast cancer (TNBC) diagnosis at or before age 60, and a male breast cancer diagnosis [1,2]. Patients with gBRCA mutations often experience more aggressive disease, with increased risk of other or secondary malignancies [2,3]. Additionally, some studies have found that BRCA mutations are associated with lower breast cancer-specific survival rates [4]. 

Due to its effect on lifetime risk of cancers, gBRCA status is a significant factor in the personalization of breast cancer treatment. A mastectomy and contralateral prophylactic mastectomy should be considered per standard of care, rather than a conservative surgery for risk reduction purposes [1,5,6]. Subsequently, this surgical decision can have downstream implications for radiation treatment [1].

Poly(ADP) ribose polymerase inhibitors (PARPi) selectively induce cell death in BRCA-mutated tumours through synthetic lethality, due to defects in the homologous recombination repair pathway in these cells [7]. Two PARPi therapies, olaparib and talazoparib, have been approved by Health Canada for use in patients with gBRCA-mutated metastatic breast cancer [8,9]. More recently, positive results were reported from the OlympiA trial, which evaluated if olaparib would provide a benefit as an adjuvant therapy in patients with early breast cancer at high risk of recurrence [1,8,10,11,12,13].

In August 2022, Health Canada approved the use of adjuvant olaparib for HER2-negative gBRCA-mutated early breast cancer, adding to the evolving armamentarium of (neo)adjuvant treatment strategies [8,14,15]. With its approval comes a new set of considerations surrounding its use in clinical practice, such as patient identification, integration with existing treatment options, and toxicity management. This opinion piece aims to discuss some of these issues, including selection of patients for hereditary cancer genetic testing, exploring strategies for the identification of high-risk disease, and navigation of new treatment pathways for TNBC and hormone receptor-positive/HER2-negative (HR+/HER2-) early breast cancer, with a specific focus on Canadian clinical practice. 

## 2. Efficacy and Safety of Olaparib in Early Breast Cancer

### 2.1. The OlympiA Trial: Key Patient Characteristics and Eligibility Criteria

OlympiA was a Phase 3, double-blinded, randomized trial evaluating the safety and efficacy of 12 months of adjuvant olaparib therapy versus placebo in high-risk, gBRCA-mutated, HER2-negative early breast cancer following definitive local treatment and adjuvant or neoadjuvant chemotherapy. Patients had completed at least six cycles of neoadjuvant or adjuvant chemotherapy containing anthracyclines, taxanes, or both agents; platinum chemotherapy was allowed. All local therapy, including radiation therapy, had to be completed at least 2 weeks and not more than 12 weeks before trial entry. In HR-positive patients, adjuvant endocrine therapy and adjuvant bisphosphonates were allowed concurrently with olaparib and administered according to institutional guidelines [13]. 

OlympiA examined four patient populations considered to have HER2-negative disease at high risk of recurrence (Table 1) [13,16]. Patients with TNBC who had received neoadjuvant therapy were required to have residual invasive breast cancer in the breast or resected lymph nodes (i.e., no pathological complete response [pCR]) to qualify, while patients with TNBC who had received adjuvant therapy were required to have axillary node-positive disease or an invasive primary tumour ≥2 cm. For patients with HR-positive disease who had received neoadjuvant therapy, risk assessment involved evaluation of their clinical-pathologic stage and nuclear grade via the CPS + EG scoring system (refer to Jeruss et al., 2008, for a detailed description of the calculation) [13,17]. These patients were considered at high risk if they had no pCR and a CPS + EG score of 3 or greater. Conversely, patients with HR-positive disease who had received adjuvant therapy were at high risk if they had at least 4 pathologically-confirmed positive lymph nodes [13]. 

### 2.2. Efficacy Outcomes in the OlympiA Trial

Overall, the OlympiA trial enrolled 1836 patients and included patients with Stage IB to IIIC disease [13]. At a pre-specified, event-driven interim analysis, treatment with olaparib was associated with a statistically significant improvement in invasive disease-free survival (IDFS), the primary endpoint, with a 3-year IDFS rate of 85.9% in the olaparib group and 77.1% in the placebo group (difference, 8.8%; stratified hazard ratio, 0.58; 99.5% confidence interval (CI), 0.41–0.82; *p* < 0.0001). Similarly, treatment with olaparib led to a significant improvement in 3-year distant disease-free survival (DDFS) (stratified hazard ratio, 0.57; 99.5% CI, 0.39–0.83; *p* < 0.0001). There was no significant difference in overall survival (OS) between the olaparib and placebo arms at this first interim analysis [13]; however, at the second interim analysis, a significant OS improvement was observed with a hazard ratio of 0.68 (98.5% CI, 0.47–0.97; *p* = 0.009, with a significance threshold of 0.015), translating to an absolute benefit of 3.4% at 4 years [16]. 

A subgroup analysis of both IDFS and OS revealed a benefit with olaparib for all stratification groups and subgroups, including both patients with TNBC (stratified hazard ratio for 4-year OS, 0.640; 95% CI, 0.459–0.884) and patients with HR-positive disease (stratified hazard ratio for 4-year OS, 0.897; 95% CI, 0.449–1.784). There was no evidence suggesting statistical heterogeneity in the treatment effect across various stratification factors, which included HR status, prior chemotherapy, prior platinum therapy, and *BRCA1/2* status [13,16]. 

### 2.3. Adverse Event Profile 

In the OlympiA trial, the most common adverse events (AEs) reported in the olaparib treatment group were gastrointestinal toxicities, fatigue, and hematologic toxicities; these were also the most common reasons for discontinuation. Notably, the only Grade 3 AE with an incidence higher than 5% was anemia, and 5.8% of patients treated with olaparib required blood transfusion versus 0.9% in the placebo group [13]. Experience from ovarian cancer suggests that upfront patient education and proactive monitoring and management of these toxicities are key for maintaining patients on olaparib [18,19,20,21,22]. 

Adverse events of special interest included pneumonitis, myelodysplastic syndrome (MDS) and acute myeloid leukemia (AML) [13]. None of these AEs of special interest occurred at a greater incidence in the olaparib group than in the placebo group over a median of 2.5 years [13]. Longer-term follow-up is required to assess the risk of MDS/AML as well as new primary cancers.

MDS is of particular interest as it is a class warning for PARPi therapies arising from trials in the ovarian cancer setting [20]. While the causes of PARPi-associated leukemogenesis remain unclear, one underlying risk factor may be previous exposure to platinum and alkylating agents, as MDS/AML has been more commonly observed in heavily pretreated patients [20,23]. The SOLO-2 trial evaluated olaparib maintenance therapy in patients with platinum-sensitive, relapsed ovarian cancer. In this setting where olaparib was taken until disease progression or discontinued at investigator discretion, the MDS/AML rate was 8% after 5 years of follow-up and a mean total duration of olaparib therapy of 29.1 months (vs. an MDS/AML rate of 4% with placebo) [24]. In the SOLO-1 trial, olaparib maintenance followed first-line platinum-based chemotherapy and was administered for a finite period of 2 years (or continued if evidence of disease per investigator discretion). After 7 years of follow-up, the MDS/AML rate was 1.5% (vs. 0.8% with placebo) [25]. 

In the breast cancer setting, the rate of MDS/AML in OlympiA was 0.1% where the planned duration of olaparib therapy was 12 months [13], and there were no cases of MDS/AML in the OlympiAD trial, in which olaparib was given for a median total treatment duration of 8.2 months in the metastatic breast cancer setting [8,26]. 

Recognizing the potential for rare but serious hematologic toxicity, patients on olaparib should be monitored routinely [8,20]. Proactive monitoring measures include baseline assessment, monthly monitoring of complete blood counts (CBC) throughout treatment, and periodic monitoring beyond 12 months [8]. Within the ovarian cancer setting, clinician opinion is that CBC should continue to be assessed every 3–4 months following completion of olaparib therapy. 

## 3. Identification of Olaparib Candidates

### 3.1. Identifying Patients at High-Risk for Recurrence

Based on the results of the OlympiA trial, the use of adjuvant olaparib in high-risk, HER2-negative early breast cancer is recommended in several guidelines and is approved by regulatory bodies in numerous countries, including Canada [6,8,27,28,29]. The Health Canada indication statement for olaparib does not define “high-risk”; however, several clinical practice guidelines recommend the selection of olaparib candidates based on the trial criteria (Table 2). Additionally, Canadian Agency for Drugs and Technologies in Health (CADTH) recommends reimbursement for olaparib under conditions that match the trial criteria (see Section 4.1 and Section 4.2) [30]. 

Notably, the OlympiA trial protocol permitted inclusion of patients with Stage IB to IIIC disease, provided the criteria for high-risk disease were met (see Table 1) [13]; however, the St. Gallen International Consensus Guideline only recommends olaparib for patients with Stage II or III disease [28]. For the purposes of adopting olaparib into Canadian clinical practice, the authors of this manuscript define high-risk early breast cancer per the OlympiA trial criteria. 

### 3.2. Hereditary Cancer Genetic Testing to Identify Olaparib Candidates

With the OlympiA trial demonstrating the efficacy benefits of olaparib in gBRCA-mutated early breast cancer, a number of guideline bodies have updated their recommendations for hereditary cancer genetic testing to facilitate testing to inform treatment decisions (see Table 2). Access to hereditary cancer genetic testing in Canada remains variable and continues to evolve with the discovery of new risk genes, targeted therapies, and improvements in genetic technologies. Some provinces and institutions have updated their criteria to provide publicly funded testing in scenarios where germline variant status will qualify a patient for an approved targeted therapy and all other criteria for that therapy are met [32]. 

Potential olaparib candidates may also be eligible for publicly funded hereditary cancer genetic testing through customary means based on personal and family history. Ontario’s most current testing criteria include individuals with a personal history of breast cancer at ≤45 years of age and triple-negative breast cancer at ≤60 years of age [32]. Unfortunately, standardized national criteria for hereditary cancer testing have not been established and Canadians face inequitable access to this pathway to personalized medicine. For now, practitioners should seek out their current regional guidance for accessing funded genetic services and become familiar with alternative genetic testing mechanisms, such as research initiatives and commercial providers for patients willing to pay for genetic testing. 

#### 3.2.1. Timing Considerations for Hereditary Cancer Genetic Testing

As gBRCA status is relevant for both surgical and adjuvant systemic treatment decisions, it is ideal for hereditary cancer genetic testing to be completed early in the treatment pathway [1,14]. Mutation status will influence the surgical options presented: a total mastectomy (uni- or bilateral) may be considered due to increased risk of a second ipsi- and/or contralateral cancer [1,14]. In scenarios where BRCA results are received after surgery and radiotherapy, a risk-reducing mastectomy may be performed later, but may negatively affect cosmetic results and increase the risk of surgical complications [1]. Accordingly, multiple guidelines suggest that germline testing be initiated as early as possible or during the workup for invasive breast cancer [6,14].

In the specific context of making PARPi treatment decisions, gBRCA status requires confirmation within the timeframe required to initiate olaparib treatment per OlympiA protocol: not more than 12 weeks after completion of local therapy, including radiation therapy [13]. In addition to testing during pre-surgical workup, a second timepoint for considering gBRCA testing exists post-surgery. If, following surgery (with or without radiotherapy) and chemotherapy, the patient is found to be at high risk for disease recurrence, then they may be a potential candidate for olaparib [6,13,27,28] (see Section 3.1 for definitions of “high risk” disease). Optimally, results of genetic testing should be obtained before any planned radiotherapy treatment since it might provide an opportunity to offer increased options for alternative risk reducing surgery in patients with clinically relevant genetic alterations. Initiating gBRCA testing following surgery requires an expedited turnaround of test results, which may be challenging in some jurisdictions. 

#### 3.2.2. Mainstreaming Genetic Testing 

Within the context of Canadian practice, current turnaround times for hereditary cancer genetic testing may pose a significant challenge for surgical planning and initiating PARPi therapy. This is especially the case when traditional testing pathways, which require upfront referral to the cancer genetics clinic for pre-test counselling, are followed; access to testing may be delayed by months. One solution to these delays is to implement mainstreamed genetic testing, a pathway in which testing is initiated by a non-genetics clinician in patient populations that meet eligibility criteria based on their own personal cancer history [33]. Beyond personal cancer history, some mainstreaming protocols may also consider family history and tumour biology. Because the specialist who first sees the patient is often a surgeon or medical oncologist in this disease setting, they are the focus of mainstreaming efforts [14,32,34]. 

Within the mainstreaming pathway, the surgeon or medical oncologist will provide pretest counselling, obtain patient consent, and directly order the genetic test [33]. In Canada, results are generally also disclosed by the ordering physician; for most patients, the cancer genetics clinic will only be involved in the consultation process when a pathogenic or likely pathogenic variant in *BRCA1/2* or other targeted genes is detected [35,36]. Depending on the region, the Genetic Services may also connect with patients when a Variant of Unknown Significance (VUS) is detected. In cases where no known variant is detected, a referral to Genetic Services is generally not required but is recommended if the patient has many questions or concerns due to personal or family history of cancers. By eliminating upfront pre-test counselling by Genetic Services, the turnaround time for testing is minimized. The cancer genetics clinic is also able to focus resources on patients who test positive, thereby easing resource constraints [34]. 

To date, mainstreamed genetic testing has been implemented in various practice sites across Canada [34,35,36]. Several strategies exist to help implement or improve efficiency in the mainstreaming pathway: screening tools can be used to help surgeons and oncologists identify patients who are eligible for testing; pre-test counselling checklists and patient handouts can support non-genetics clinicians in educating patients and obtaining consent in a 5–10 min timeframe [37]. Standardized results letters and phone calls can also be used to disclose results to patients, and some locales have mechanisms in place to reflexively refer patients to the cancer genetics clinic whenever a positive result is obtained [33,35,36,38]. Mainstreaming requires cross-departmental coordination, and any site seeking to establish a mainstreaming pathway should do so in collaboration with their local cancer genetics service [32,35]. 

## 4. Treatment Pathways for HER2-Negative Early Breast Cancer

### 4.1. Adjuvant Treatment Options for Early High-Risk TNBC

Three key adjuvant therapy trials inform treatment choice in the early, high-risk TNBC patient population: CREATE-X, which assessed capecitabine versus standard therapy in patients with residual disease after NACT; KEYNOTE-522, which assessed the addition of neoadjuvant/adjuvant pembrolizumab versus placebo; and OlympiA for olaparib (see Table 3) [13,39,40]. The KEYNOTE-522 and OlympiA trials supported Health Canada approvals for the use of adjuvant pembrolizumab and olaparib in high-risk early breast cancer, respectively [8,41]; however, a Health Canada-approved indication was not pursued for capecitabine [42]. Despite this, most provinces in Canada fund capecitabine for early breast cancer based on the CREATE-X trial (see Table 3).

**Figure 1:**
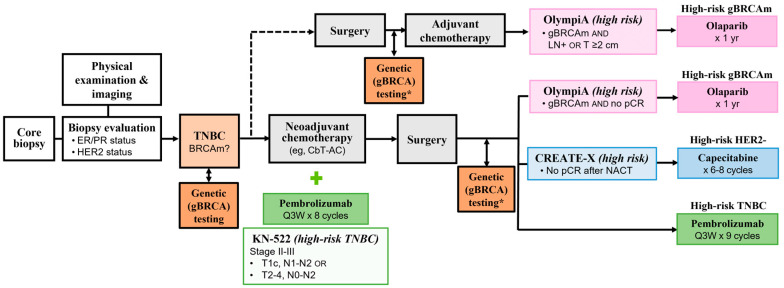
Treatment pathways for early, high-risk TNBC, with adjuvant therapy options and the “high risk” definitions used in their respective pivotal trials highlighted in pink (olaparib), blue (capecitabine), or green (pembrolizumab). For TNBC, the neoadjuvant chemotherapy pathway is more frequently pursued within the current standards of care; surgery followed by adjuvant chemotherapy is a less common pathway (dotted line). BRCAm, BRCA-mutated; CbT-AC, carboplatin and paclitaxel/doxorubicin or epirubicin and cyclophosphamide; ER, estrogen receptor; gBRCAm, germline BRCA-mutated; LN, lymph node; NACT, neoadjuvant chemotherapy; pCR, pathological complete response; PR, progesterone receptor; Q3W, every 3 weeks. * If not done earlier.

When assessing the eligibility of a patient for any of these three adjuvant options, it is imperative to understand the differences between the study populations enrolled in their respective pivotal trials, the high-risk disease definition used by each trial, and the timing of these treatments relative to chemotherapy and surgery (Table 3, Figure 1). Prescribers should also note the duration of these new therapies and the potential toxicities endured by patients during extended adjuvant therapies. Notably, all patients enrolled in OlympiA carried a germline BRCA mutation [13]. Conversely, KEYNOTE-522 and CREATE-X did not mandate genetic testing and outcomes related to this specific subgroup of patients were not reported in either trial. While it is helpful to note these differences between the pivotal trials, it is also critical to avoid cross-trial comparisons, as pre-specified endpoints and statistical plans are different between them all. 

### 4.2. Adjuvant Treatment Options for High-Risk HR+/HER2- Early Breast Cancer

**Figure 2:**
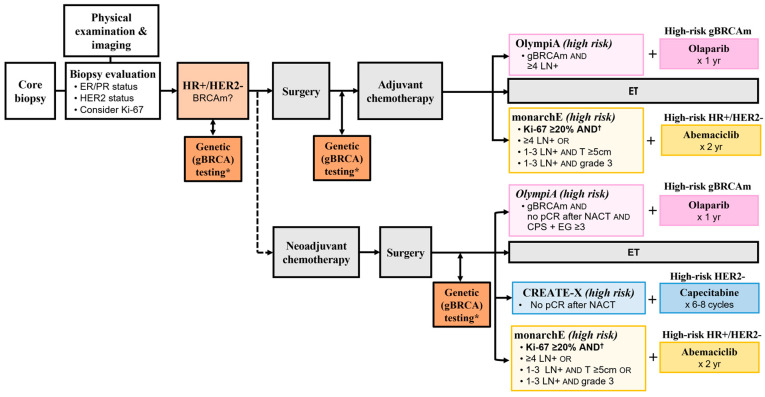
Treatment pathways for high-risk HR+/HER2- early breast cancer. “High-risk disease” criteria for the OlympiA trial (pink), CREATE-X trial (blue), and CADTH recommendation for abemaciclib (yellow) are overlayed. In HR+/HER2- disease, surgery followed by adjuvant chemotherapy is commonly pursued within the current standards of care; the neoadjuvant chemotherapy pathway is less common (dotted line). ER, estrogen receptor; ET, endocrine therapy; BRCAm, *BRCA* mutation; CADTH, Canadian Agency for Drugs and Technologies in Health; gBRCA, germline *BRCA*; gBRCAm, germline *BRCA*-mutated; HR, hormone receptor; LN, lymph node; NACT, neoadjuvant chemotherapy; pCR, pathological complete response; PR, progesterone receptor. * If not done earlier. † For abemaciclib: The CADTH reimbursement recommendation requires Ki-67 ≥20% and specific clinicopathological features per monarchE Cohort 1. Cohort 2 did not receive a funding recommendation [56].

Adjuvant therapy options for high-risk, early-stage HR+/HER2- disease include capecitabine, the CDK4/6 inhibitor (CDK4/6i) abemaciclib, and olaparib; safety and efficacy for these therapies were assessed in the CREATE-X, monarchE, and OlympiA Phase 3 trials, respectively (Table 4, Figure 2) [13,39,55]. Olaparib and abemaciclib both have Health Canada approval for this indication, while capecitabine, although funded in most provinces for high-risk HER2-negative early breast cancer, does not (see Table 4). As stated previously, while it is critical to note key differences between the pivotal trials when selecting a treatment option, it is also imperative to avoid cross-trial comparisons, as pre-specified endpoints and statistical plans differ between the trials. 

The CREATE-X trial included both HR-positive and TNBC patients and demonstrated a disease-free survival benefit in its intention-to-treat population (Table 4); a subgroup analysis showed a greater benefit in TNBC patients (HR, 0.58; 95% CI, 0.39–0.87) and less promising results in HR-positive patients (DFS HR, 0.81; 95% CI, 0.55–1.17; *n* = 601) [39]. Because of its uncertain benefit in HR-positive disease, as well as the existence of other adjuvant treatment options, physicians tend not to consider capecitabine in HR-positive early breast cancer. Within the OlympiA trial, which also included both HR-positive and TNBC patients, only 18% of the intention-to-treat population had HR-positive disease. Accordingly, the CADTH reimbursement recommendation for olaparib noted that due to the small sample size of patients with HR-positive disease, there is uncertainty with the results of its HR-positive subgroup analysis (OS HR, 0.897; 95% CI, 0.449–1.784; *n* = 325 patients) [16,30]. 

In the HR+/HER2- early breast cancer setting, there are varying definitions for “high-risk disease”. Notably, for abemaciclib, the Health Canada indication and CADTH reimbursement recommendation specify a requirement for Ki-67 ≥20% regardless of lymph node involvement (see Table 4) [56,57]. The CADTH reimbursement recommendation further limits funded use of abemaciclib in early breast cancer to patients with features aligned with Cohort 1 and Ki-67 ≥20% [56]; an overview of this monarchE pre-specified analysis was published by Royce and colleagues [29]. Interestingly, although the U.S. Food and Drug Administration (FDA) previously also required a Ki-67 score ≥20% for the approved use of abemaciclib, this requirement was removed in March 2023 [58,59]. The decision followed a 4-year interim analysis update demonstrating that the IDFS benefit of abemaciclib is not dependent on Ki-67 index [60]. Given these data, as well as the decision by the FDA, there is potential for an update to the Health Canada indication in the future. 

The Health Canada indication for olaparib does not include the detailed definitions of high-risk disease presented in the OlympiA trial [8,13]. However, the CADTH reimbursement recommendation states that olaparib should be reimbursed in patients who meet OlympiA trial criteria (Table 4); various clinical practice guidelines similarly recommend the use of olaparib in patients meeting OlympiA eligibility criteria [6,27,28,30]. 

**Table 4:** Key Adjuvant Therapy Trials in Patients with High-Risk HR+/HER2- Early Breast Cancer, Health Canada, and CADTH Guidance

	Capecitabine|CREATE-X (*n* = 910) [39]	Abemaciclib|monarchE (*n* = 5367) [55]	Olaparib|OlympiA (*n* = 1836) [13]
Population	Stage I-III, HER2-negative BC - TNBC or HR+/HER2- No pCR (breast and/or nodes) after NACT	HR+/HER2- high-risk eBC after surgery and RT and/or AdjCT/NACT	gBRCA-mutated HER2-negative early breast cancer TNBC or HR+/HER2- Received local treatment + NACT or AdjCT x 6 wk with anthracyclines, taxanes, or both
Definition of “High Risk” per Trial Criteria	No pCR, after NACT containing anthracycline, taxane, or both	**Cohort 1** ≥4 LN+ or 1–3 LN+ *and* ≥1 of the following: - T ≥5 cm - Grade 3 **Cohort 2** 1–3 LN+ *and* Ki-67 ≥20%	HR+/HER2- *^†^ If AdjCT: ≥4 LN+ If NACT: No pCR with a CPS + EG score ≥3
Intervention	**Capecitabine** 1250 mg/m^2^ PO BID days 1–14 Q3W x 6–8 cycles **Control arm:** standard therapy	**Abemaciclib** (150 mg BID x ≤2 years) +SOC ET (5–10 years as clinically indicated) **Control arm:** SOC ET (5–10 years as clinically indicated)	**Olaparib** 300 mg PO BID x 1 year **Control arm:** Placebo PO x 1 year
Primary Endpoint	**ITT Population: HER2-** Median follow-up: 3.6 years [39] **3-year DFS:** 82.8 vs. 73.9%; Δ 8.9% **5-year DFS:** 74.1 vs. 67.6%; Δ 6.5% **DFS HR:** 0.70 (95% CI, 0.53–0.92); *p* = 0.01	**ITT Population: HR+/HER2-** Median follow-up: 15.5 months [55] **2-year IDFS:** 92.2 vs. 88.7%; Δ 3.5% **IDFS HR:** 0.75 (95% CI, 0.60–0.93; *p* = 0.01) Median follow-up: 42 months [61] **4-year IDFS**: 85.8% vs. 79.4%; Δ 6.4% **IDFS HR:** 0.664 (95% CI, 0.578–0.762)	**ITT Population: HER2-** Median follow-up: 2.5 years [13] **3-year IDFS:** 85.9 vs. 77.1%; Δ 8.8% **IDFS HR:** 0.58 (99.5% CI, 0.41–0.82); *p* < 0.0001 Median follow-up: 3.5 years [16] **4-year IDFS:** 82.7% vs. 75.4%; Δ 7.3% **IDFS HR:** 0.63 (95% CI, 0.50–0.78)
Exploratory Subgroup Analysis of DFS/IDFS	**Subgroup: ER+ or PgR+** Median follow-up: 3.6 years [39] **DFS:** 76.4% vs. 73.4%; Δ 3.0% **DFS HR:** 0.81 (95% CI, 0.55–1.17) *p*-value for HR status interaction: 0.21 (NS)	**CADTH Population: ^‡^ Cohort 1, Ki-67 ≥20%** [29,56] Median follow-up: 27 months **IDFS HR:** 0.63 (95% CI, 0.49–0.80)	**Subgroup: HR+/HER2-** Median follow-up: 2.5 years [13] **3-year IDFS:** 83.5 vs. 77.2% **IDFS HR:** 0.70 (95% CI, 0.38–1.27) Heterogeneity tests: NS Median follow-up: 3.5 years [16,44] **4-year IDFS:** 80.1% vs. 76.6%; Δ 3.5% **IDFS HR:** 0.680 (95% CI, 0.402–1.134) *p*-value for heterogeneity: 0.754 (NS)
Secondary Endpoint: OS	**ITT Population: HER2-**Median follow-up: 3.6 years [39] **5-year OS:** 89.2 vs. 83.6%; Δ 5.6% **OS HR:** 0.59 (95% CI, 0.39–0.90)	**ITT Population: HR+/HER2- **Median follow-up: 27 months [55] **ITT, OS HR:** 1.091 (95% CI, 0.818–1.455) Median follow-up: 42 months [61] **ITT, OS HR:** 0.929 (95% CI, 0.748–1.153); *p* = 0.50	**ITT Population: HER2-**Median follow-up: 3.5 years [16] **4-year OS:** 89.8 vs. 86.4% (ITT); Δ 3.4% **OS HR:** 0.68 (98.5% CI, 0.47–0.97); *p* = 0.009 ^§^
Exploratory Subgroup Analysis of OS	**Subgroup: ER+ or PgR+**Median follow-up: 3.6 years [39] **OS:** 93.4% vs. 90.0%; ∆ 3.4% **OS HR:** 0.73 (95% CI, 0.38–1.40) *p*-value for HR status interaction: 0.41 (NS)	**CADTH Population: ^‡^ Cohort 1, Ki-67 ≥20%** [29,56] Median follow-up: 27 months **OS HR**: 0.767 (95% CI, 0.511–1.152)	**Subgroup: HR+/HER2-**Median follow-up: 3.5 years [16,44] **4-year OS**: 88.1% vs. 86.3% ∆ 1.8% **OS HR:** 0.897 (95% CI, 0.449–1.784) *p*-value for heterogeneity: 0.381 (NS)
Health Canada Indication and CADTH Recommendation	No indication for early breast cancer approved by Health Canada [42] Funded in most provinces [45,46,47,48,49,50,51,52,53]	**Health Canada:** [57] In combination with ET for the adjuvant treatment of HR+/HER2-, LN+ **eBC at high risk** of disease recurrence **based on clinicopathological features and a Ki-67 score ≥20%** **CADTH Recommended Population:** [56] HR+/HER2- eBC with Ki-67 ≥20% *and* one of the following: - ≥4 LN+ - 1–3 LN+ *and* Grade 3 - 1–3 LN+ *and* T ≥ 5	**Health Canada:** [8] Patients with **gBRCA-mutated, HER2-negative high risk eBC** who have been treated with neoadjuvant or adjuvant chemotherapy **CADTH Recommended Population:** [30] Per OlympiA trial criteria (Section 2.1, Table 1)

* Risk assessment was performed at the time of surgery. ^†^ The OlympiA trial included patients with both TNBC and HR+/HER2- breast cancer. Only high-risk HR+/HER2- disease criteria are shown here. For high-risk TNBC criteria, refer to Table 3. ^‡^ A gated hierarchical testing strategy included IDFS in patients with a Ki-67 score ≥20% from cohort 1 alone [29]. ^§^ Significance boundary of 0.015. Summary of key trials for adjuvant capecitabine, abemaciclib, and olaparib in high-risk, HR+/HER2- early breast cancer. AdjCT, adjuvant chemotherapy; BID, *bis in die* (twice daily); CADTH, Canadian Agency for Drugs and Technologies in Health; DFS, disease-free survival; eBC, early breast cancer; HR, hazard ratio; HR+/HER2-, hormone receptor-positive/human epidermal growth factor receptor 2-negative; IA1/2, interim analysis 1 or 2; ITT, intention-to-treat; LN, lymph node; NACT, neoadjuvant chemotherapy; NS, not significant; Q3W, every 3 weeks; RT, radiation therapy; SOC ET, standard of care endocrine therapy.

Figure 2 depicts the possible treatment pathways for HR+/HER2- disease, including these three adjuvant treatment options and the varying definitions for high-risk disease that are relevant to Canadian practice. Note that risk criteria in the OlympiA trial varied depending on the timing of chemotherapy: for patients receiving neoadjuvant therapy, a CPS + EG score was used to assess their risk; for patients without neoadjuvant therapy, the requirement was disease in 4 pathologically confirmed lymph nodes. Additionally, of the three trials, only OlympiA required the presence of a gBRCA mutation [8,13].

### 4.3. Considerations for Sequencing Olaparib with Other Therapies in the Adjuvant Setting

#### 4.3.1. Olaparib, Radiation, and Endocrine Therapy

With the introduction of new adjuvant treatment options, clinicians are seeking guidance on the sequencing or combining of therapies. Adjuvant olaparib can be given concurrently with endocrine therapy, consistent with the protocol in the OlympiA trial [6,13]. In cases where radiation is indicated, it is common for radiation therapy to follow chemotherapy. Olaparib must be given at least 2 weeks after completion of radiation therapy, as PARP inhibition has a known radiosensitizing effect [6,13,62]. Additionally, per the OlympiA trial protocol, olaparib therapy should be initiated within 12 weeks of completion of the last treatment, which may include surgery, radiation, or chemotherapy [13]. With regard to timing, the CADTH reimbursement recommendation notes that some situations may warrant treatment initiation beyond this 12-week timeframe for certain patients with high-risk breast cancer, such as legacy patients [30].

#### 4.3.2. Olaparib and Other Adjuvant Treatment Options 

Currently, the NCCN Guidelines^®^ suggest that the sequential or combined use of pembrolizumab, olaparib, and/or capecitabine may be considered in select patients with a high risk of recurrence and who meet criteria for treatment with one of more of these agents, although the guidelines also state that there are presently no data on sequencing or combining adjuvant pembrolizumab with olaparib in patients [6]. The absence of combination data represents a key knowledge gap in the treatment of HER2-negative early breast cancer, and various ongoing trials are evaluating the efficacy and safety of concurrent therapies with PARPi treatments including olaparib (Table 5). 

##### Olaparib and Immunotherapy

With recent approvals of both immunotherapy and PARPi treatment in TNBC [8,41,63], there is notable interest in the feasibility of combining these two drug classes. While limited, there is published experience with olaparib in combination with pembrolizumab in patients with breast cancer (see Table 5). Outcomes from these studies suggest that efficacy is unaltered, and that patient toxicity is acceptable with a manageable safety profile. Notably, the ongoing phase II/III KEYLYNK-009 study is evaluating the clinical benefit of pembrolizumab plus olaparib maintenance therapy after first-line chemotherapy with pembrolizumab in locally recurrent inoperable or metastatic TNBC [64]. Results from this study, as well as other trials focusing on sequential/combination therapies, will inform the integration of olaparib with immune-oncology therapies in routine practice. 

##### Olaparib and Abemaciclib

Our search of the literature and ClinicalTrials.gov registry revealed one ongoing National Cancer Institute trial investigating olaparib in combination with abemaciclib in recurrent ovarian cancer (see Table 5). This dose escalation study is examining concurrent use of these two agents. In this early phase of clinical adoption of olaparib therapy, clinicians have expressed substantial concern regarding the potential cumulative toxicity of this combination; it is anticipated that oncologists will choose either abemaciclib or olaparib, giving consideration to their respective toxicity profiles and duration of therapy. Although the survival benefit observed in the OlympiA trial was in a study population in which only 18% of patients had HR-positive breast cancer, the monarchE trial has not yet reached maturity for its OS analysis. This currently translates in many physicians having a clinical preference for prescribing olaparib for gBRCA-mutated, HR-positive patients [16,55,59].

**Table 5:** Select Clinical Trials of Olaparib/PARPi Combination Therapy in Breast Cancer and Other Solid Tumours*.

Trial	Population	Intervention	Outcomes
**Olaparib and Pembrolizumab in Breast Cancer**			
**KEYLYNK-0072** [65] (NCT04123366) Phase II, single-arm, open-label study	Previously treated advanced solid tumours with mutations in homologous recombination repair genes *and/or* homologous recombination deficiency (including breast cancer) (N = 168)	Olaparib 300 mg BID + pembrolizumab 200 mg IV Q3W (35 cycles) until PD or unacceptable AEs	Grade 3/4 TRAEs, 35.7%; grade 5 TRAEs, 0 Discontinuations due to TRAEs, 2,4% Common TRAEs: nausea, 39.3%; anemia, 30.4%; fatigue, 15.5% Authors noted that “olaparib + pembrolizumab showed promising antitumour activity with manageable safety…”
**TOPACIO/KEYNOTE-162 ^†^** [66] (NCT02657889) Phase II, single arm, open-label study	Advanced/metastatic TNBC (irrespective of BRCA status or PD-L1 expression) (N = 55)	Niraparib 200 mg PO daily^†^ + pembrolizumab 200 mg IV Q3W	Most common grade ≥3 AEs: anemia, 18%; thrombocytopenia, 15%; fatigue, 7% IRAEs: any, 15%; grade 3, 2% Authors noted that the treatment showed “promising antitumour activity” and a “tolerable safety profile”
**KEYLYNK-009** [64] (NCT04191135) Phase II/III, randomized, open-label study	Locally recurrent inoperable or metastatic TNBC (estimated N = 932)	Induction pembrolizumab + carboplatin-gemcitabine chemotherapy Maintenance with: - Pembrolizumab 200 mg Q3W + olaparib 300 mg BID; *or* - Pembrolizumab + chemotherapy	Trial ongoing
**NCT05203445** [67] Phase II single-arm, open-label study	Newly diagnosed TNBC or HR+/HER2- BC (N = 23)	Olaparib 300 mg BID + pembrolizumab 400 mg IV Q6W (x 12 weeks) followed by chemotherapy and surgery	Trial ongoing
**Olaparib and Pembrolizumab in Other Solid Tumours**			
**KEYLYNK-010** [68] (NCT03834519) Phase III, randomized, open-label study	mCRPC (molecularly unselected) (N = 793)	Arms: Pembrolizumab 200 mg IV Q3W for ≤35 cycles + olaparib 300 mg PO BID Abiraterone or enzalutamide daily	Grade ≥3 TRAEs, 35% vs. 9% Grade ≥3 IMAEs, 5% vs. 1% Authors noted that “While pembrolizumab + olaparib resulted in more grade ≥3 TRAEs vs. NHA in patients with previously treated mCRPC, no new safety signals occurred… [69]” “Most common AEs were anemia, nausea, fatigue, and decreased appetite [69]”
**KEYNOTE-365** [70] (NCT02861573) Phase Ib/II, non-randomized, multicohort, open-label study (Cohort A)	mCRPC (molecularly unselected) (Cohort A: N = 102)	Cohort A: Pembrolizumab 200 mg IV Q3W olaparib 400 mg tab or 300 mg cap PO BID	Authors noted a “safety profile consistent with the profiles of the individual agents and demonstrated antitumor activity”
**ENGOT-OV43/KEYLYNK-001** [71] (NCT03740165) Phase III, randomized, double-blind study	1L ovarian cancer (BRCA non-mutated) (N = 1367)	Arms: CbT Q3W x 5 cycles + pembrolizumab 200 mg IV Q3W x up to 35 cycles + olaparib 300 mg PO BID starting cycle 7 CbT Q3W x 5 cycles + pembrolizumab 200 mg IV Q3W x up to 35 cycles + placebo PO BID CbT Q3W x 5 cycles + placebo IV Q3W + placebo PO BID	Trial ongoing
**KEYLYNK-012** [72] (NCT04380636) Phase III, randomized, placebo- and active-controlled, double-blind study	Unresectable stage III NSCLC (N = 870)	Arms: Pembrolizumab + CRT followed by pembrolizumab + placebo Pembrolizumab + CRT followed by pembrolizumab + olaparib CRT followed by durvalumab	Trial ongoing
**KEYLYNK-013** [73] (NCT04624204) Phase III, randomized, double-blind study	Limited-stage SCLC (N = 672)	Arms: Pembrolizumab + CRT followed by pembrolizumab + placebo Pembrolizumab + CRT followed by pembrolizumab + olaparib Pembrolizumab + CRT followed by placebo	Trial ongoing
**Olaparib and Abemaciclib in Solid Tumours**			
**NCI-2020-10084** [74] (NCT04633239) Phase I/Ib, open-label, dose escalation study	Recurrent ovarian cancer (N = 42)	Olaparib PO BID on days 1–28 + abemaciclib PO BID on days 8–28 of cycle 1 and days 1–28 of subsequent cycles Cycles repeat every 28 days in the absence of disease progression or unacceptable toxicity	Trial ongoing

* Based on a non-systematic search of medical literature and ClinicalTrials.gov based on the following keywords: “olaparib” or “PARP inhibitor” + “pembrolizumab”, “abemaciclib”, or “capecitabine”. ^†^ Note: Niraparib clinical trial included. AE, adverse events; BID, *bis in die* (twice daily); cap, capsules; CbT, carboplatin-paclitaxel; CRT, chemoradiotherapy; HRD, homologous recombination deficiency; HRRm, homologous recombination repair mutation; IMAE, immune-mediated adverse events; IRAE, immune-related adverse events; IV, intravenous; mCRPC; metastatic castration-resistant prostate cancer; NHA, next-generation hormonal agent; NSCLC, non-small cell lung cancer; PD, progressive disease; PD-L1, programmed death ligand 1; PO, *per os* (orally); Q3W, every 3 weeks; SCLC, small cell lunger cancer; tabs, tablets; TRAE, treatment-related adverse events; TNBC, triple-negative breast cancer.

##### Olaparib and Capecitabine

There is a paucity of information regarding the sequential use or combination of olaparib with capecitabine [6]. Like abemaciclib, there is concern surrounding potential cumulative toxicity from combined use of capecitabine and olaparib, and it is probable that many oncologists will choose one over the other in practice. Some physicians are also considering sequential use of capecitabine followed by olaparib in patients with high-risk, gBRCA-mutated TNBC, although there are no data to support this strategy. 

## 5. Conclusions

The recent approval of olaparib in Canada for HER2-negative early breast cancer offers a novel option for personalized treatment of gBRCA-mutated, high-risk early breast cancers. This new indication for olaparib presents a need for early determination of gBRCA status to facilitate systemic therapy planning, as well as surgical decision-making and familial risk identification. Mainstreaming led by oncologists or surgeons offers a potential path to streamlined, patient-centered genetic testing to ensure that results are received in time for treatment decisions. In addition to gBRCA status, the identification of high-risk disease is also critical to personalizing care for patients with HER2-negative breast cancer, as multiple adjuvant therapy options are available for both high-risk TNBC and high-risk HR+/HER2- disease. Capecitabine, olaparib, and pembrolizumab are notable options for high-risk TNBC, whereas abemaciclib, capecitabine, and olaparib are options for high-risk HR+/HER2- disease. Selection between these adjuvant treatments should be guided by the patient’s germline BRCA status and the respective criteria for high-risk disease. For patients who are eligible for multiple treatment options, however, there are very limited data to guide the selection, sequencing, or combination of these therapies. 

Furthermore, recent data presented at the 2023 ASCO Annual Meeting demonstrated that another CDK4/6i regimen, ribociclib with endocrine therapy, shows an IDFS benefit in the adjuvant setting for Stage IB-III early breast cancer [75]. This emerging option may be incorporated into future guidelines and/or algorithms but has not received approval from either the FDA or Health Canada at the time of this publication. PARPi combinations are being explored in a variety of solid tumours, which may provide insights into the safety of these treatment regimens. However, few of these studies focus specifically on early breast cancer, highlighting a need for more trials in this disease setting. Any future trials or real-world evidence examining the combination or sequencing of these therapies, or the comparative efficacy or safety of these treatment options, will provide useful information for evolving the clinical management of early-stage, HER2-negative breast cancer.

## Figures and Tables

**Table 1:** High-risk Patient Populations in the OlympiA Trial [13].

HER2-Negative Disease	Prior Therapy	High-Risk Criteria
TNBC	Neoadjuvant	Non-pCR
	Adjuvant	≥pT2 or ≥pN1
HR-positive	Neoadjuvant	Non-pCR and CPS + EG score ≥3 *
	Adjuvant	≥4 LN+

* Refer to Jeruss et al., 2008 for CPS + EG score calculation [17]. CPS + EG, clinical stage, pathologic stage, ER status, and tumour grade; HR, hormone receptor; LN, lymph node; pCR, pathological complete response; TNBC, triple-negative breast cancer.

**Table 2:** Guideline Recommendations for Olaparib Eligibility in Early Breast Cancer.

Guideline	Recommendation for Olaparib Eligibility	Recommendation for gBRCA Testing to Inform Treatment Decisions
The American Society of Clinical Oncology (ASCO)	One year of adjuvant olaparib for patients with early-stage, gBRCA-mutated, HER2-negative cancer with a high risk of recurrence after completion of (neo)adjuvant chemotherapy and local treatment, including radiation. “High risk” is defined as the four patient subpopulations that were eligible for the OlympiA trial (see Section 2, Table 1) [27].	n/a
NCCN Clinical Practice Guidelines in Oncology (The NCCN Guidelines^®^)	One year of adjuvant olaparib should be considered for patients with gBRCA-mutated HER2-negative disease who fall into the four high-risk populations enrolled in OlympiA [6].	In addition to other personal and family history criteria, testing should be done whenever it will aid adjuvant treatment decisions with olaparib in high-risk, HER2-negative breast cancer [6,31].
2021 St. Gallen International Consensus Guidelines	Adjuvant olaparib for patients with Stage II or III HER2-negative disease meeting OlympiA trial criteria (support from >93% panelists), or patients with Stage II or III HER2-negative cancers regardless of estrogen receptor status or prior treatment with platinum-based chemotherapy (support from 64% of panelists) [28].	gBRCA testing is recommended for patients meeting the OlympiA trial criteria in order to identify candidates for olaparib therapy [28].

gBRCA, germline *BRCA*; HER2, human epidermal growth factor 2.

**Table 3:** Key Adjuvant Therapy Trials in Patients with Early High-Risk TNBC, Health Canada, and CADTH Guidance.

	Capecitabine|CREATE-X (*n* = 910) [39]	Pembrolizumab|KEYNOTE-522 (*n* = 1174) [40]	Olaparib|OlympiA (*n* = 1836) [13]
Population	Stage I-III, HER2-negative BC - TNBC or HR+/HER2- No pCR (breast and/or nodes) after NACT	Stage II/III TNBC Any PD-L1 status	gBRCA-mutated HER2-negative eBC - TNBC or HR+/HER2- Received local treatment + NACT or AdjCT x 6 weeks with anthracyclines, taxanes, or both
Definition of “High Risk” per Trial Criteria	No pCR after NACT containing anthracycline, taxane, or both	T1c, N1-2; T2-4 N0-N2 *	**TNBC ^†‡^** If AdjCT: LN+ or pT ≥2 cm If NACT: No pCR after NACT
Intervention	**Capecitabine** 1250 mg/m^2^ PO BID days 1–14 Q3W x 6–8 cycles **Control arm:** standard therapy	**Experimental arm:** NACT: [**Pembrolizumab** Q3W + Cb + T] x 4 cycles then [**Pembrolizumab** Q3W + A + C] x 4 cycles Surgery then AdjTx [**Pembrolizumab** Q3W x 9 cycles] **Placebo arm:** NACT: [Placebo + Cb + T] x 4 cycles then [Placebo + A + C] x 4 cycles Surgery then AdjTx [Placebo Q3W x 9 cycles]	**Olaparib** 300 mg PO BID x 1 year Placebo PO x 1 year
Primary Endpoint	**ITT Population: HER2-** Median follow-up: 3.6 years [39] **3-year DFS:** 82.8% vs. 73.9%; Δ 8.9% **5-year DFS:** 74.1% vs. 67.6%; Δ 6.5% **DFS HR:** 0.70 (95% CI, 0.53–0.92); *p* = 0.01	**ITT Population: TNBC** Median follow-up: 15.5 months [40] **pCR (ypT0/Tis ypN0):** 64.8% vs. 51.2%; Δ 13.6% **18-month EFS:** 91.3% vs. 85.3%; Δ 6.0% **EFS HR:** 0.63 (95% CI, 0.43–0.93) Median follow-up: 39.1 months [43] **3-year EFS:** 84.5% vs. 76.8%; Δ 7.7% **EFS HR:** 0.63 (95% CI, 0.48–0.82); *p* < 0.001	**ITT Population: HER2-** Median follow-up: 2.5 years [13] **3-year IDFS:** 85.9% vs. 77.1%; Δ 8.8% **IDFS HR:** 0.58 (99.5% CI, 0.41–0.82); *p* < 0.0001 Median follow-up: 3.5 years [16] **4-year IDFS:** 82.7% vs. 75.4%; Δ 7.3% **IDFS HR:** 0.63 (95% CI, 0.50–0.78)
Exploratory Subgroup Analyses of DFS/IDFS	Subgroup: ER- and PgR- Median follow-up: 3.6 years [39] **DFS:** 69.8% vs. 56.1%; Δ 13.7 **DFS HR:** 0.58 (95% CI, 0.39–0.87) *p*-value for HR status interaction: 0.21	*n/a*	Subgroup: TNBC Median follow-up: 2.5 years [13] **3-year IDFS:** 86.1% vs. 76.9% **IDFS HR:** 0.56 (95% CI, 0.43–0.73) Heterogeneity tests: NS Median follow-up: 3.5 years [16,44] **4-year IDFS:** 83.1% vs. 75.2%; Δ 7.9% **IDFS HR:** 0.620 (95% CI, 0.487–0.787) *p*-value for heterogeneity: 0.754 (NS)
Secondary Endpoint: OS	ITT Population: HER2- Median follow-up: 3.6 years [39] **5-year OS:** 89.2% vs. 83.6%; Δ 5.6% **OS HR:** 0.59 (95% CI, 0.39–0.90)	ITT Population: TNBC Median follow-up: 39.1 months [43] **3-year OS:** 89.7% vs. 86.9%; Δ 2.8% ^§^ **OS HR:** 0.72 (95% CI, 0.51–1.02) ^§^	ITT Population: HER2- Median follow-up: 3.5 years [16] 4-year OS: 89.8% vs. 86.4%; Δ 3.4% OS HR: 0.68 (98.5% CI, 0.47–0.97); *p* = 0.009 ^‖^
Exploratory Subgroup Analyses of OS	Subgroup: ER- and PgR- Median follow-up: 3.6 years [39] **OS:** 78.8% vs. 70.3%; Δ 8.5% **OS HR:** 0.52 (95% CI, 0.30–0.90) *p*-value for HR status interaction: 0.41	***n/a***	Subgroup: TNBC Median follow-up: 3.5 years [16,44] 4-year OS: 90.1% vs. 86.3%; Δ 4.8% OS HR: 0.640 (95% CI, 0.459–0.884) *p*-value for heterogeneity: 0.381 (NS)
Health Canada Indication and CADTH Recommendation	No indication for early breast cancer approved by Health Canada [42] Funded in most provinces [45,46,47,48,49,50,51,52,53]	**Health Canada:** [41] **High-risk early-stage TNBC** in combination with chemotherapy as neoadjuvant treatment, and then continued as monotherapy as adjuvant treatment after surgery **CADTH Recommended Population:** [54] Per KEYNOTE-522 trial criteria	**Health Canada:** [8] Patients with **gBRCA-mutated, HER2-negative high risk eBC** who have been treated with neoadjuvant or adjuvant chemotherapy **CADTH Recommended Population:** [30] Per OlympiA trial criteria (Section 2.1, Table 1)

* According to the AJCC, 7th edition. ^†^ Risk assessment was performed at the time of surgery. ^‡^ The OlympiA trial included patients with both TNBC and HR+/HER2- breast cancer. Only high-risk TNBC criteria are shown here. For high-risk HR+/HER2- disease criteria, refer to Table 4. ^§^ Not significant. ^‖^ Significance boundary of 0.015. Summary of key trials for adjuvant capecitabine, pembrolizumab, and olaparib in early-stage, high-risk TNBC. A, doxorubicin or epirubicin; AdjCT, adjuvant chemotherapy; AdjTx, adjuvant treatment; BID, *bis in die* (twice daily); C, cyclophosphamide; CADTH, Canadian Agency for Drugs and Technologies in Health; Cb, carboplatin; DFS, disease-free survival; eBC, early breast cancer; EFS, event-free survival; ER, estrogen receptor; HR, hazard ratio; HR+/HER2-, hormone receptor-positive/human epidermal growth factor receptor 2-negative; IA1/2, interim analysis 1 or 2; IDFS, invasive disease-free survival; ITT, intention-to-treat; LN, lymph node; N, node; NACT, neoadjuvant chemotherapy; NS, not significant; OS, overall survival; pCR, pathological complete response; PgR, progesterone receptor; PD-L1, programmed death ligand 1; PO, *per os* (orally); Q3W, every 3 weeks; T, paclitaxel; TNBC, triple-negative breast cancer.

## Data Availability

No new data were created or analyzed in this study. Data sharing is not applicable to this article.
